# Hepatic enzyme ALT as a marker of glucose abnormality in men with cystic fibrosis

**DOI:** 10.1371/journal.pone.0219855

**Published:** 2019-07-18

**Authors:** Johann Colomba, Silvia R. Netedu, Catherine Lehoux-Dubois, Adèle Coriati, Valérie Boudreau, François Tremblay, Kenneth Cusi, Rémi Rabasa-Lhoret, Julio A. Leey

**Affiliations:** 1 Montreal Clinical Research Institute (IRCM), Montréal, Québec, Canada; 2 Department of Nutrition, Faculty of Medicine, Université de Montréal, Montréal, Québec, Canada; 3 Department of Medicine, Faculty of Medicine, Université de Montréal, Montréal, Québec, Canada; 4 Adult Cystic Fibrosis Centre, Department of Respirology, St. Michael’s Hospital, Toronto, Ontario, Canada; 5 Cystic Fibrosis Clinic, Centre hospitalier de l’Université de Montréal (CHUM), Montréal, Québec, Canada; 6 Division of Endocrinology, Diabetes and Metabolism, University of Florida, Gainesville, Florida, United States of America; University of the Chinese Academy of Sciences, CHINA

## Abstract

**Aim:**

Cystic fibrosis (CF) patients are at high risk of developing CF-related diabetes (CFRD). In non-CF patients, liver disease, specifically steatosis and non-alcoholic fatty liver disease (NAFLD), is strongly associated with type 2 diabetes. We compared glycemic status and metabolic profiles in CF patients according to a biomarker of hepatic injury, alanine aminotransferase (ALT).

**Methods:**

We conducted a cross-sectional study among 273 adult CF patients recruited from the Montreal CF Cohort. A 2-hour oral glucose tolerance test (OGTT) was performed to collect glucose and insulin measures every 30 minutes. Fasting ALT levels and anthropometric measures were also obtained. Patients were categorized into 2 groups based on ALT cut-off of 25 U/L.

**Results:**

Patients in the high ALT group were mostly men (83%), had higher mean weight and BMI (p<0.001) and showed elevated glucose levels throughout OGTT (p≤0.01). When stratified by sex, only men with high ALT showed significantly higher weight (p<0.001), higher glycemic values at 60, 90 and 120 minutes of OGTT (p≤0.01), higher frequency of *de novo* CFRD (20.5% vs 8.2%, p = 0.04) as well as lower insulin sensitivity than men with normal ALT (p = 0.03). ALT levels were strongly associated with HOMA-IR in CFRD patients (p = 0.001, r^2^ = 0.28).

**Conclusions:**

Adult CF men with higher ALT show an increased frequency of dysglycemia and *de novo* CFRD, lower insulin sensitivity and higher eight. Our data suggests that ALT levels could be an interesting tool to guide targeted diabetes screening, particularly among CF men. Prospective studies are needed to confirm these observations.

## Introduction

There is increasing evidence of a strong association between liver disease, particularly hepatic steatosis and non-alcoholic fatty liver disease (NAFLD), and the presence of type 2 diabetes mellitus (T2DM) [1, 2], with insulin resistance as a main factor involved [3]. Even lean patients with NAFLD are typically insulin resistant [4]. Plasma measures of alanine aminotransferase (ALT) and aspartate aminotransferase (AST) are routinely used to detect hepatocellular damage. Although they can be normal in NAFLD patients, several studies have shown that high ALT levels, but not AST, can predict the risk for T2DM and are also associated with insulin resistance [5–7].

Cystic fibrosis (CF) is a genetic disease involving a mutation in the CF transmembrane conductance regulator (*CFTR*) gene which results in accumulation of viscous secretions affecting primarily the respiratory and digestive systems [8]. The pancreas is often severely affected, leading to exocrine pancreatic insufficiency, malabsorption and low weight [9]. Endocrine pancreatic failure is also frequently encountered, with CF-related diabetes (CFRD) being now one of the most common complications of CF, especially in the context of prolonged life expectancy of this population [10]. The cause of CFRD is multifactorial and a decreased beta-cell mass with decreased insulin secretion is recognized as being the main factor underlying the pathophysiology [11–13]. The role of insulin resistance is currently debated, but there is evidence to suggest its contribution to the development of dysglycemia in CF patients, particularly in certain states, such as the use of steroids and acute pulmonary infections [11, 14]. Furthermore, it has been shown that in the context of low insulin secretion, insulin resistance can modulate glucose tolerance changes over time [15, 16] and in response to exercise [17].

CF has been associated with various hepatic abnormalities, ranging from liver transaminitis and steatosis to secondary biliary cirrhosis and portal hypertension [18]. Although there is no clear consensus on the definition of abnormal liver enzyme levels in CF, the use of lower thresholds has been proposed in order to detect initial signs of liver damage. Pediatric liver centers now recognize ALT >25 U/L for boys and ALT >22 U/L for girls as the threshold for abnormal ALT in CF patients [18]. Hepatic steatosis can develop in CF patients in response to malnutrition and uncontrolled CFRD [19]. It is not clear though if it increases the probability of progression towards fibrosis and cirrhosis, as it is observed in the non-CF population [18]. However, a recent retrospective chart review study identified 17 out of 114 CF patients (14.9%) with hepatic steatosis based on abdominal imaging [20]. These patients with liver steatosis had higher HbA1c, higher body mass index (BMI) and higher levels of ALT, supporting an association between liver steatosis and dysglycemia [20].

The purpose of our study was to further investigate the potential association between liver disease and dysglycemia in CF, by comparing glucose tolerance status and metabolic profiles in adult CF patients according to ALT level, the main plasma biomarker of hepatic injury. Moreover, we wanted to assess the response in men and women separately, given that sex differences have already been documented by our group in CF in terms of insulin secretion and insulin sensitivity [21].

## Material and methods

### Patients

Participants were recruited from the Montreal CF cohort (MCFC), a prospective observational cohort designed to study glucose intolerance development and its association with clinical outcomes. Patients are followed every 12 to 18 months and they undertake on the same day an oral glucose tolerance test (OGTT), pulmonary function tests and a detailed medical examination [21–23]. The MCFC main inclusion criteria are adult age (≥18 years old), confirmed CF diagnosis and not known CFRD. Exclusion criteria are previous diagnosis of diabetes, ongoing pregnancy, pulmonary exacerbation in the previous month, conditions that could interfere with glucose metabolism such as intravenous antibiotics, corticosteroids (oral or intravenous) or growth hormone treatment and previous lung or liver transplantation [21]. Patients categorized as *de novo* CFRD following the OGTT protocol were referred to an endocrinologist to undergo further tests to confirm the diagnosis. All patients in the MCFC who have available data for OGTT plasma glucose and insulin and also ALT levels at baseline were included in the analysis.

### Clinical data

Age, sex and genotype were obtained from medical files. Patients on pancreatic enzymes replacement supplements were categorized as having pancreatic insufficiency. Body weight and fat mass were determined using an electronic scale (Tanita Corporation Arlington Heights, IL, USA) and standing height was measured using a wall stadiometer. BMI was calculated using weight in kilograms divided by height in squared meter (kg/m^2^). Pulmonary capacity was measured by spirometry on the same day as the OGTT and was expressed using the percentage of predicted forced expiratory volume in 1 second (FEV_1_%: Medgraphic 1870, ST Paul, MN, USA).

### Oral glucose tolerance test (OGTT) and categories for glucose tolerance

Patients had to fast for 8 hours and then consume a sweet glucose beverage (1.75g/kg of body weight up to a maximum of 75g or 300 ml) in less than 5-min. A catheter was installed before the test for blood sampling [24]. Blood samples were taken at 0, 30, 60, 90 and 120-min of the OGTT to measure glycemic excursion and insulin levels over time. Plasma glucose was analysed by Glucose Analyzer (YSI 2300 STAT plus, glucose and lactate analyzing; YSI Inc.). Insulin samples were frozen at -80°C then chemically measured by Immunitubidimeter, ADVIAI650; Bayer Health Car diagnosis.

Based on their 2-hours glucose value during the OGTT, subjects were categorized as having normal glucose tolerance (NGT; <7.8 mmol/L), impaired glucose tolerance (IGT; ≥ 7.8 mmol/L and <11.1 mmol/L), indeterminate glucose tolerance (INDET; <7.8 mmol/L, but 1-h glycemia ≥11.1 mmol/L) or de novo CFRD (≥11.1 mmol/L). Fasting glucose value ≥7.0 mmol/L was also considered as de novo CFRD. Insulin sensitivity was calculated using the Stumvoll index and HOMA-IR formula as previously defined [15, 25].

### Blood sample data

Fasting blood sample was also used to measure hepatic enzymes (ALT, AST, GGT), lipid profile and glycosylated hemoglobin (HbA1c), as described previously [21]. All hepatic enzymes measurements were done in the same laboratory in order to avoid inter-assay variability. As no widely accepted ALT cut-off exists in CF population to predict the development of liver disease, we relied on previous proposed data from CF [18] and non-CF patients [6, 26] and set the ALT cut-off at 25 U/L in our analysis.

### Statistical analysis

The results are presented as mean ± standard deviation (SD). A student's t-test was performed to compare groups’ means. A Chi-square was performed for categorical variables. Pearson’s correlation was used to assess the association between ALT level and HOMA-IR. All analyses were performed on SPSS 17.0 program for Windows (SPPS, Chicago, IL). Statistical tests were considered significant at p-value <0.05.

## Results

A total of 273 patients were included in the study (Table 1). The mean age of the population was 25.7 ± 8.0 years, the mean BMI was 21.8 ± 3.0 kg/m^2^ and the mean FEV1 was 73.3 ± 21.8%. Pancreatic insufficiency was present in 79.3% of the patients. There were more patients with pancreatic insufficiency in the high ALT group (85.7%) than in the normal ALT group (75.3%). When we examined the overall population, we observed a high prevalence of men in the high ALT group compared to the normal ALT group (83% vs 36.5%, p < 0.001). We also noticed a higher BMI in the high ALT group (23.0 ± 3.0 kg/m^2^ vs 21.0 ± 2.6 kg/m^2^, p <0.001). Interestingly, the FEV_1_% was not significantly different between the 2 groups (p = 0.332).

**Table 1:** Baseline patient characteristics and metabolic profile comparison between normal ALT and high ALT groups.

	All patients N = 273	Normal ALT (< 25 U/L) N = 167	Hight ALT (≥ 25 U/L) N = 106	*p*-value
Age (years)	25.7 ± 8.0	25.1 ± 8.1	26.8 ± 8.0	0.085
Sex (% men)	54.6	36.5	83.0	< 0.001*
ALT (U/L)	25.3 ± 14.6	17.3 ± 4.1	38.0 ± 16.0	**< 0.001**
**Glucose tolerance group**				
NGT (%)	39.9	42.5	35.8	0.177*
IGT (%)	28.9	30.5	26.4	0.177*
INDET (%)	16.8	16.2	17.9	0.177*
CFRD (%)	14.3	10.8	19.8	0.177*
DelF508 mutation (% homozygote)	47.8	45.2	55.3	0.160*
Pancreatic Enzyme Supplement (%)	79.3	75.3	85.7	0.045*
FEV_1_ (%)	73.3 ± 21.8	72.7 ± 21.8	75.3 ± 21.2	0.332
Weight (kg)	60.3 ± 11.1	56.9 ± 9.3	66.2 ± 11.3	**< 0.001**
BMI (kg/m^2^)	21.8 ± 3.0	21.0 ± 2.6	23.0 ± 3.0	**< 0.001**
Fat mass (%)	18.9 ± 7.8	19.4 ± 8.1	18.3 ± 7.4	0.247
**OGTT glucose measure (mmol/L)**				
T0 min	5.5 ± 0.8	5.3 ± 0.7	5.7 ± 0.9	**< 0.001**
T30 min	9.3 ± 1.8	9.3 ± 1.8	10.1 ± 2.2	**0.001**
T60 min	10.2 ± 3.1	10.7 ± 2.6	12.2 ± 3.6	**< 0.001**
T90 min	9.9 ± 3.5	9.1 ± 2.8	11.0 ± 4.2	**< 0.001**
T120 min	8.0 ± 3.3	7.6 ± 2.8	8.6 ± 3.8	**0.013**
AUC_0-120min_	1121.9 ± 284.7	1063.1 ± 225.6	1213.1 ± 339.3	**< 0.001**
**OGTT insulin measure (μU/mL)**				
T0 min	10.6 ± 5.1	10.7 ± 5.2	10.5 ± 5.0	0.718
T30 min	33.7 ± 22.5	34.3 ± 21.7	32.6 ± 24.0	0.556
T60 min	54.2 ± 38.5	57.2 ± 41.3	49.7 ± 33.9	0.143
T90 min	59.4 ± 39.2	63.4 ± 41.8	53.0 ± 34.7	**0.047**
T120 min	51.8 ± 38.5	55.5 ± 40.5	45.7 ± 34.9	0.052
AUC_0-120min_	5326.6 ± 3196.6	5591.4 ± 3331.1	4901.1 ± 2986.1	0.102
HbA1c (%)	5.7 ± 0.6	5.7 ± 0.5	5.8 ± 0.6	**0.007**
Insulin Sensitivity (Stumvoll index)	0.072 ± 0.027	0.073 ± 0.026	0.071 ± 0.030	0.618
HOMA-IR	2.58 ± 1.28	2.53 ± 1.23	2.66 ± 1.36	0.441
AST (mmol/L)	25.6. ± 8.1	20.1 ± 4.3	31.1 ± 12.0	**< 0.001**
GGT (mmol/L)	19.7 ± 18.5	15.0 ± 13.0	27.2 ± 23.0	**< 0.001**
TG (mmol/L)	1.2 ± 0.8	1.2 ± 0.6	1.2 ± 1.0	0.792
CHOL (mmol/L)	3.5 ± 0.9	3.6 ± 1.0	3.4 ± 0.9	0.158
HDL (mmol/L)	1.2 ± 0.3	1.2 ± 0.3	1.1 ± 0.3	**0.023**
LDL (mmol/L)	1.8 ± 0.7	1.8 ± 0.8	1.8 ± 0.7	0.642

ALT: alanine aminotransferase; NGT: normal glucose tolerance; IGT: impaired glucose tolerance; INDET: indeterminate glucose tolerance; CFRD: cystic fibrosis-related diabetes; FEV1: forced expiratory volume expired in 1 second; BMI: body mass index; OGTT: oral glucose tolerance test; AUC0-120min: area under the curve from T0 to T120 min OGTT; HbA1c: glycosylated hemoglobin; HOMA-IR: insulin resistance index, AST: aspartate aminotransferase, GGT: gamma-glutamyl transferase; TG: triglycerides; CHOL: cholesterol; HDL: high-density lipoprotein cholesterol, LDL: low-density lipoprotein cholesterol.

Data are presented as mean ± SD. Student’s t-test was performed to compare groups’ means.

*Chi square test was performed for categorical variables. Statistical significance was set at *p* ≤ 0.05. Values in bold represent significant *p*-values.

**Fig 1:**
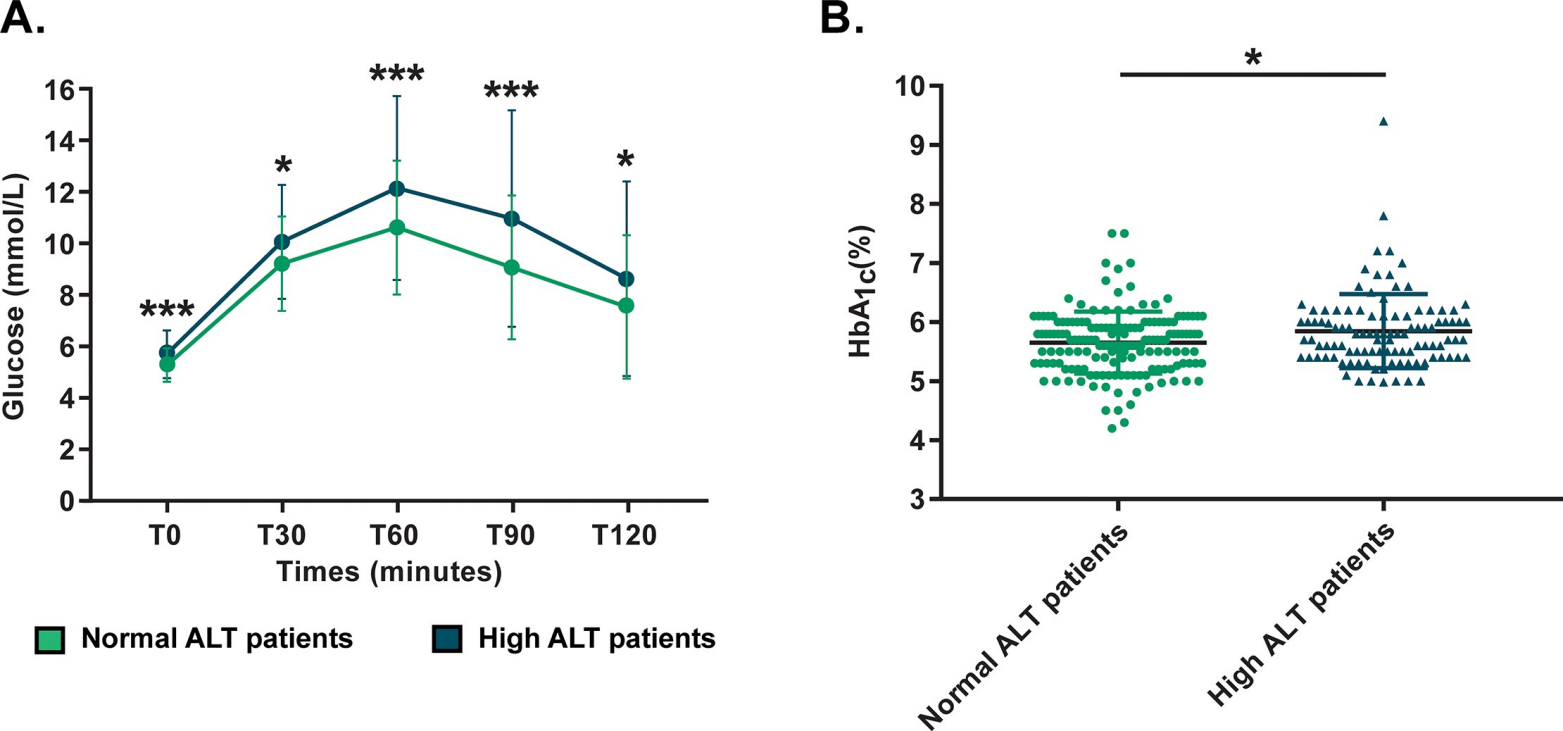
**Glycemia excursion during OGTT (A) and HbA1c (B) in normal and high ALT groups.** Patients in high ALT group demonstrate higher glycemia levels at all OGTT time-points and higher HbA1c. *p ≤ 0.05; ***: *p* ≤ 0.001.

The high ALT group showed more elevated glucose levels for all OGTT time-points (p ≤0.013) (Fig 1), without significant changes in insulin levels overall. Although it remained in the normal range, HbA1c was also higher in the high ALT group (5.8 ± 0.6% vs 5.7 ± 0.5%, p = 0.007). Even though it didn’t reach statistical significance, high ALT group CF patients had a 9% absolute increase in CFRD incidence (19.8% vs 10.8%, p = 0.177). Furthermore, HDL cholesterol levels were slightly lower in the high ALT group (1.14 ± 0.29 mmol/L vs 1.23 ± 0.33 mmol/L, p = 0.023). We also found a statistical difference in AST and GGT values between the normal and high ALT groups (p <0.001), but only ALT showed association with the T120-min OGTT glycemia (r = 0.129, p = 0.033), which was not observed with the other liver enzymes.

A sub-analysis of the CFRD group (n = 39) revealed that CFRD patients with high ALT had higher glycemia levels for all OGTT time-points (p = 0.037), higher values of AUC_Gluc-total_ (p = 0.005), higher HOMA-IR (p = 0.040) and lower insulin sensitivity by Stumvoll index (p = 0.045) than CFRD patients with normal ALT (Table 2). There also was a strong positive correlation between ALT and HOMA-IR (p = 0.001, r = 0.527), which represents 27.8% of the variance. This association didn’t exist for AST or GGT.

**Table 2:** Comparison of glycemic profile between normal ALT and high ALT in CFRD patients.

	All CFRD patients N = 39	Normal ALT (< 25 U/L) N = 18	High ALT (≥ 25 U/L) N = 21	*p*-value
Age (years)	26.4 ± 7.2	25.4 ± 7.9	27.2 ± 6.7	0.403
Sex (% men)	59.0	27.8	85.7	< 0.001*
ALT (U/L)	29.0 ± 13.2	18.1 ± 3.6	38.4 ± 11.0	**< 0.001**
**OGTT glucose measure (mmol/L)**				
T0 min	6.3 ± 1.2	5.8 ± 0.8	6.6 ± 1.4	**0.037**
T30 min	11.4 ± 2.6	10.4 ± 1.8	12.2 ± 3.0	**0.027**
T60 min	15.5 ± 3.4	14.0 ± 2.6	16.7 ± 3.6	**0.011**
T90 min	15.9 ± 3.5	14.2 ± 2.1	17.3 ± 3.9	**0.004**
T120 min	14.3 ± 2.5	13.3 ± 2.5	15.1 ± 2.3	**0.031**
AUC_0-120min_	1592.1 ± 398.9	1445.2 ± 208.1	1713.0 ± 337.5	**0.005**
HbA1c (%)	6.33 ± 0.82	6.26 ± 0.63	6.40 ± 0.97	0.602
Insulin sensitivity (Stumvoll index)	0.037 00B1 0.024	0.046 ± 0.021	0.030 ± 0.25	**0.045**
HOMA-IR	3.07 ± 1.62	2.49 ± 1.32	3.59 ± 1.72	**0.040**

ALT: alanine aminotransferase; OGTT: oral glucose tolerance test; AUC0-120min: area under the curve from T0 to T120 min OGTT; HbA1c: glycosylated hemoglobin; HOMA-IR: insulin resistance index.

Data are presented as mean ± SD. Student’s t-test was performed to compare groups’ means.

*Chi square test was performed for categorical variables. Statistical significance was set at *p* ≤ 0.05. Values in bold represent significant *p*-values.

**Fig 2:**
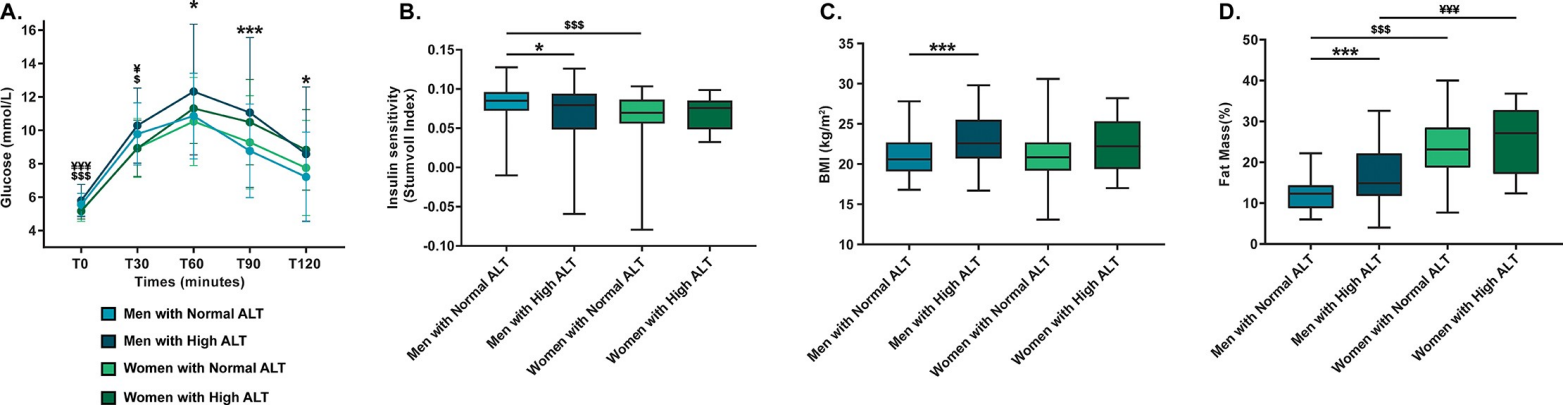
**Glucose (A), insulin sensitivity (B), BMI (C) and fat mass percentage (D) differences between normal and high ALT groups following sex stratification.** Men with high ALT have higher glucose levels at T60 to T120 min and show markers of decreased insulin sensitivity compared to men with normal ALT. Men with high ALT have higher BMI and higher percentage of fat body mass than men with normal ALT. No significant differences were observed between the women groups. *: *p* ≤ 0.05 between men with normal and high ALT; ***: *p* ≤ 0.001 between men with normal and high ALT; ^$^: *p* ≤ 0.05 between men and women with normal ALT; ^$ $ $^: *p* ≤ 0.001 between men and women with normal ALT; ^¥^: *p* ≤ 0.05 between men and women with high ALT; ^¥¥¥^: *p* ≤ 0.001 between men and women with high ALT.

When stratified by sex, we observed that mostly men showed significant differences for the majority of glycemic and metabolic variables at study (Table 3). Men in the high ALT group presented higher glucose values for OGTT time-points T60-min to T120-min (p ≤0.014) (Fig 2A). They were also more frequently affected by CFRD (20.5% vs 8.2%, p = 0.04). A significantly lower insulin sensitivity by Stumvoll index (p = 0.029) was also found in men with high ALT compared to those with normal ALT (Fig 2B). Additionally, the high ALT group demonstrated a higher mean body weight (68.3 ± 10.6 kg vs 61.5 ± 9.1 kg, p <0.001) and BMI (23.2 ± 3.0 kg/m^2^ vs 21.0 ± 2.3 kg/m^2^, p <0.001) (Fig 2C and 2D).

**Table 3:** Metabolic profile comparison between normal ALT and high ALT groups following sex stratification.

	Men (N = 149)			Women (N = 124)			Men vs. Women *p*-value	
	Normal ALT (< 25 U/L) n = 61	High ALT (≥ 25 U/L) n = 88	*p*-value	Normal ALT (< 25 U/L) n = 106	High ALT (≥ 25 U/L) n = 18	*p*-value	Normal ALT (< 25 U/L)	High ALT (≥ 25 U/L)
Age (years)	24.7 ± 7.1	26.7 ± 8.2	0.123	25.3 ± 8.6	27.3 ± 7.2	0.352	0.639	0.770
ALT (U/L)	18.3 ± 3.6	37.5 ± 14.9	**< 0.001**	16.6 ± 4.2	40.4 ± 21.3	**< 0.001**	0.009	0.484
CFRD (%)	5 (8.2)	18 (20.5)	0.042*	13 (12.3)	3 (16.7)	0.606*	0.417	0.717
**Glucose tolerance group**			0.244*			0.660*	0.417	0.717
NGT (N/%)	27 (44.3)	33 (37.5)		44 (41.5)	5 (27.8)			
IGT (N/%)	16 (26.2)	20 (22.7)		35 (33.0)	8 (44.4)			
INDET (N/%)	13 (21.3)	17 (19.3)		14 (13.2)	2 (11.1)			
CFRD (N/%)	5 (8.2)	18 (20.5)		13 (12.3)	3 (16.7)			
Pancreatic enzymes (%)	78.3	88.5	0.095	73.6	72.2	0.904	0.496	0.072
FEV1 (%)	72.5 ± 24.0	75.3 ± 21.3	0.467	72.8 ± 20.7	75.3 ± 20.9	0.633	0.947	0.997
Weight (kg)	61.5 ± 9.1	68.3 ± 10.6	**< 0.001**	54.3 ± 8.4	55.8 ± 8.4	0.473	**< 0.001**	**< 0.001**
BMI (kg/m^2^)	21.0 ± 2.3	23.2 ± 3.0	**< 0.001**	21.0 ± 2.8	22.4 ± 3.3	0.067	0.897	0.328
Fat mass (%)	12.3 ± 4.1	16.8 ± 6.3	**< 0.001**	23.4 ± 7.0	25.6 ± 8.4	0.225	**< 0.001**	**< 0.001**
**OGTT glucose measure (mmol/L)**								
T0 min	5.6 ± 0.7	5.8 ± 1.0	0.090	5.2 ± 0.6	5.2 ± 0.5	0.915	**< 0.001**	**< 0.001**
T30 min	9.8 ± 1.9	10.3 ± 2.2	0.124	9.0 ± 1.8	8.9 ± 1.7	0.965	**0.004**	**0.014**
T60 min	10.9 ± 2.6	12.4 ± 3.8	**0.005**	10.5 ± 2.6	11.3 ± 2.1	0.238	0.437	0.113
T90 min	8.8 ± 2.8	11.1 ± 4.5	**< 0.001**	9.3 ± 2.8	10.5 ± 2.6	0.088	0.260	0.588
T120 min	7.2 ± 2.7	8.6 ± 4.0	**0.014**	7.8 ± 2.9	8.8 ± 2.4	0.132	0.227	0.730
AUC_0-120min_	1074.6 ± 211.9	1229.6 ± 361.2	**0.001**	1056.5 ± 233.8	1132.3 ± 187.9	0.195	0.619	0.104
**OGTT insulin measure (μU/mL)**								
T0 min	9.9 ± 5.4	10.8 ± 5.4	0.387	11.2 ± 5.1	9.2 ± 2.9	0.122	0.167	0.248
T30 min	29.8 ± 21.6	33.4 ± 25.5	0.412	36.8 ± 21.4	29.1 ± 15.6	0.156	0.061	0.507
T60 min	50.4 ± 41.4	49.5 ± 35.1	0.894	61.1 ± 41.0	50.7 ± 28.5	0.322	0.136	0.889
T 90 min	52.6 ± 30.1	52.0 ± 35.7	0.918	69.2 ± 46.0	58.0 ± 30.0	0.353	**0.023**	0.529
T120 min	41.4 ± 28.0	43.0 ± 33.8	0.786	63.3 ± 44.2	58.0 ± 38.2	0.648	**0.002**	0.108
AUC_0-120min_	4755.4 ± 2804.0	4853.5 ± 3091.8	0.855	6044.2 ± 3515.5	5119.5 ± 2514.0	0.302	**0.024**	0.741
HbA1c (%)	5.6 ± 0.6	5.8 ± 0.7	**0.041**	5.7 ± 0.5	5.9 ± 0.4	0.056	0.471	0.632
Insulin sensitivity (Stumvoll index)	0.082 ± 0.023	0.072 ± 0.032	**0.029**	0.068 ± 0.026	0.069 ± 0.021	0.870	**0.001**	0.727
HOMA-IR	2.47 ± 1.35	2.78 ± 1.44	0.221	2.57 ± 1.16	2.13 ± 0.72	0.138	0.648	0.075
AST (mmol/L)	20.8 ± 3.9	31.5 ± 12.5	**< 0.001**	19.8 ± 4.5	29.4 ± 9.5	**< 0.001**	**0.015**	0.510
GGT (mmol/L)	18.4 ± 19.5	28.9 ± 24.2	**0.004**	13.1 ± 6.2	18.7 ± 14.3	0.132	**0.011**	0.096
TG (mmol/L)	1.1 ± 0.6	1.2 ± 1.1	0.704	1.2 ± 0.6	1.1 ± 0.6	0.513	0.731	0.643
CHOL (mmol/L)	3.3 ± 1.0	3.3 ± 0.8	0.687	3.8 ± 0.9	3.9 ± 1.1	0.611	**0.001**	**0.009**
HDL (mmol/L)	1.1 ± 0.3	1.1 ± 0.3	0.814	1.3 ± 0.3	1.4 ± 0.3	0.252	**< 0.001**	**< 0.001**
LDL (mmol/L)	1.7 ± 0.8	1.8 ± 0.6	0.406	1.9 ± 0.7	2.0 ± 0.8	0.723	**0.019**	0.141

ALT: alanine aminotransferase; NGT: normal glucose tolerance; IGT: impaired glucose tolerance; INDET: indeterminate glucose tolerance; CFRD: cystic fibrosis-related diabetes; FEV1: forced expiratory volume expired in 1 second; BMI: body mass index; OGTT: oral glucose tolerance test; AUC0-120min: area under the curve from T0 to T120 min OGTT; HbA1c: glycosylated hemoglobin; HOMA-IR: insulin resistance index, AST: aspartate aminotransferase, GGT: gamma-glutamyl transferase; TG: triglycerides; CHOL: cholesterol; HDL: high-density lipoprotein cholesterol, LDL: low-density lipoprotein cholesterol.

Data are presented as mean ± SD. Student’s t-test was performed to compare groups’ means.

*Chi square test was performed for categorical variables. Statistical significance was set at p ≤ 0.05. Values in bold represent significant *p*-values.

For the high ALT women compared to normal ALT women, we noticed a trend towards higher BMI (p = 0.067), higher glycemia at T90-min (p = 0.097) and also higher Hb1Ac (p = 0.056), but those differences remained statistically non-significant. Also, there was no ALT-dependent difference in pulmonary function (FEV_1_%) for either sex.

## Discussion

CF patients are at increased risk of developing glucose intolerance that could eventually progress to CFRD [10, 12]. CF patients are also at higher risk for liver disease [18, 19]. In the general population, fatty liver disease is a strong risk factor for dysglycemia [1, 2, 5]. We report here the association between glucose tolerance status and easily accessible biomarkers of hepatic function in a CF population cohort. Our results demonstrate that higher ALT levels are associated with higher glycemic levels, higher HbA1c and more frequent de novo CFRD diagnosis. These observations were particularly obvious in CF men. High ALT levels are similarly associated to higher weight and BMI and lower insulin sensitivity, which supports the hypothesis that CFRD development might share metabolic pathways frequently observed in T2DM. Thus ALT measures could help in better targeting adult patients for glucose intolerance screening by annual OGTT. Interestingly, the presence of higher ALT and worse glycemic control didn’t affect pulmonary capacity, as evaluated by FEV_1_%, which is a key clinical outcome in CF patients.

Our results revealed that CF patients with higher ALT levels have higher weight and BMI, particularly in men. It is an interesting trend, as previous data have shown a predominance of adult men with CF as being overweight or obese [11]. We have also observed an increase in body fat in high ALT men, although we couldn’t specifically quantify the fat distribution in our cohort. Insulin resistance is closely related to visceral fat deposition which induces low-grade inflammation and oxidative stress and predisposes to liver injury and NAFLD [3, 27]. A previous study has shown that CF patients also have increased central fat distribution compared to healthy controls, which could influence insulin sensitivity [28]. Half of these patients were diagnosed with diabetes. Our results also point towards lower insulin sensitivity in the group of men with high ALT when measured by Stumvoll index, but not with HOMA-IR. Although no insulin sensitivity index has been thoroughly validated in CF cohorts, the Stumvoll index has been used previously by our group because of its capacity to screen for dynamic change in a glucose tolerance challenge [15, 21], while the HOMA-IR mostly reflects fasting insulin resistance. When we underwent a sub-analysis focusing on CFRD patients only, we found that the metabolic profile seems to be worse in those with ALT levels ≥ 25 U/L. Such patients had worse glycemic values at all OGTT time-points and evidence of increased estimated insulin resistance, when estimated by both Stumvoll index and HOMA-IR. This data suggests that a mechanism of hepatic insulin resistance induced by steatosis could play a role in the increase of plasma glucose concentration even in CF patients. Furthermore, ALT levels were strongly correlated with insulin resistance indices. This supports our hypothesis that high ALT status, indicative of hepatic damage, is associated with dysglycemia and incidence of CFRD through a mechanism probably implicating insulin resistance.

Early recognition and treatment of CFRD is important, given that its occurrence is associated with adverse outcomes in terms of increase risk for weight loss and pulmonary function decline leading to a higher early mortality, but also because of the risk of diabetes-related microvascular complications (e.g. retinopathy) [12, 24]. The current gold standard for CFRD screening is an annual 75-g oral glucose tolerance test (OGTT) [24] which can be a substantial burden for patients and care providers [29]. Given the low adherence of patients to OGTT screening for CFRD [30], having another simple screening tool that can be used conjointly, such as hepatic ALT, becomes interesting in order to target patients and encourage them to follow the recommended screening guidelines, especially in the younger patient population. Also, it might be worth evaluating if a combination of ALT measures with fructosamine or HbA1c in CF patients is robust enough to screen for CFRD. This way, it could reduce the number of required and hopefully avoid repetitive OGTT and ease the screening process in the CF population.

Despite similar trends the metabolic parameters were not significantly different between the two hepatic enzyme female groups. Among reasons for such sex difference could include the fact the we had a small group of women with high ALT (n = 18) this group size might limit our power. In addition, it is possible that appropriate cut-off could be gender specific as some studies suggest that normal values are lower in women [31, 32]. In addition, adult women with CF can have a preserved insulin secretion [21] which is a key factor to preserve normal glucose regulation, it has been speculated that estrogens can modulate insulin secretion through several actions on pancreatic β-cell function [21, 33]. It is also possible that well known body fat distribution between sexes with at least up to menopause a far less tendency for visceral fat deposition in women could also explain a lower risk for diabetes and hepatic steatosis in some women [34].

Our data suggests that men with higher ALT tend to accumulate factors associated to the metabolic syndrome. It is worth mentioning that the mean age of our population is less than 30 years old, which could mean that with advancing age observed abnormalities could tend to increase. Indeed recent data suggest that with aging the cause of metabolic profile could evolve [35].

Our study has some limitations. It might be difficult to generalize the data, given the single center analysis with a mostly French-Canadian ethnic origin. Still, this large cohort is well-characterized and comparable for key parameters, such as weight and pulmonary function, to other North-American published data [36]. Given the cross-sectional design, we can only comment on associations between the variables at study. On the other hand, our findings are relevant because hepatic enzymes are low cost and easily accessible biomarkers, though they only give a crude assessment of liver damage without an indication on the actual cause of liver damage. We are confident though that by choosing a lower ALT threshold in accordance to available literature, our data revealed an association that has been repeatedly observed before in T2DM. Also, it is important to note that in cross-sectional studies, biochemical parameters can change along the follow-up period.

## Conclusions

In summary, this study suggests a potential new use of ALT in CF, as a simple biomarker which is associated with dysglycemia, predominantly in men. If our results are confirmed in other patient group, ALT could help in targeting the frequency of OGTT testing as the proposed cut-off at 25 U/L allowed to detect twice as more men with CFRD. To our knowledge, this is the first study to describe glycemic variation and insulin sensitivity in relation to ALT levels in a CF population, supporting similarities between the development of CFRD and T2DM. Additional studies are required to confirm these observations and validate the most appropriate ALT cut-off to use in clinical practice.
